# *catena*-Poly[1-[(2-fluoro­benzyl­idene)amino]­quinolinium [plumbate(II)-tri-μ-iodido]]

**DOI:** 10.1107/S1600536811051853

**Published:** 2011-12-07

**Authors:** Hai-Rong Zhao

**Affiliations:** a School of Biochemical and Environmental Engineering, Nanjing Xiaozhuang College, Nanjing 210017, People’s Republic of China

## Abstract

The title complex, {(C_16_H_12_FN_2_)[PbI_3_]}_*n*_, consists of 1-[(2-fluoro­benzyl­idene)amino]­quinolinium cations and a polymeric PbI_3_^−^ anion formed by face-sharing PbI_6_ octa­hedra. These octa­hedra form straight and regular infinite chains along the *b* axis. In the asymmetric unit, one cation and one anionic [PbI_3_]^−^ fragment are observed in general positions. Polymeric chains are produced by the glide plane perpendicular to the *a* axis.

## Related literature

For second-order non-linear optical (NLO) properties, pyroelectricity, ferroelectricity and triboluminescence of inorganic-organic hybrid materials, see: Guloy *et al.* (2001 ▶); Horiuchi *et al.* (2010 ▶); Chen *et al.* (2001 ▶). For related structures, see: Bi *et al.* (2008 ▶); Zhang *et al.* (2006 ▶); Duan *et al.* (2011 ▶); Zhao *et al.* (2010 ▶).
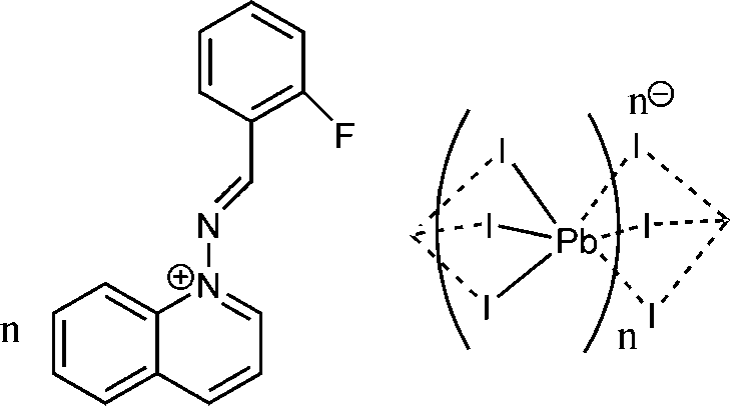


## Experimental

### 

#### Crystal data

(C_16_H_12_FN_2_)[PbI_3_]
*M* *_r_* = 839.17
Orthorhombic, 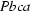

*a* = 20.888 (4) Å
*b* = 7.9112 (15) Å
*c* = 25.197 (5) Å
*V* = 4163.8 (14) Å^3^
*Z* = 8
Mo *K*α radiation
μ = 12.56 mm^−1^
*T* = 296 K
0.04 × 0.02 × 0.01 mm

#### Data collection

Siemens SMART CCD area-detector diffractometer
Absorption correction: multi-scan (*SADABS*; Sheldrick, 2002 ▶) *T*_min_ = 0.747, *T*_max_ = 0.882
30847 measured reflections
4090 independent reflections
1770 reflections with *I* > 2σ(*I*)
*R*_int_ = 0.156

#### Refinement

*R*[*F* ^2^ > 2σ(*F* ^2^)] = 0.047
*wR*(*F* ^2^) = 0.082
*S* = 0.96
4090 reflections
208 parameters
H-atom parameters constrained
Δρ_max_ = 0.96 e Å^−3^
Δρ_min_ = −0.95 e Å^−3^

### 

Data collection: *SMART* (Siemens, 1996 ▶); cell refinement: *SAINT* (Siemens, 1996 ▶); data reduction: *SAINT*; program(s) used to solve structure: *SHELXS97* (Sheldrick, 2008 ▶); program(s) used to refine structure: *SHELXL97* (Sheldrick, 2008 ▶); molecular graphics: *SHELXTL* (Sheldrick, 2008 ▶); software used to prepare material for publication: *SHELXTL*.

## Supplementary Material

Crystal structure: contains datablock(s) global, I. DOI: 10.1107/S1600536811051853/im2337sup1.cif

Structure factors: contains datablock(s) I. DOI: 10.1107/S1600536811051853/im2337Isup2.hkl

Additional supplementary materials:  crystallographic information; 3D view; checkCIF report

## Figures and Tables

**Fig. 1.**
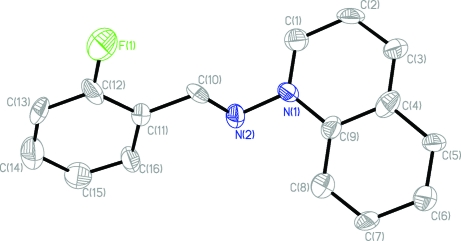
Molecular structure of the cation showing displacement ellipsoids at the 30% probability level.

**Fig. 2.**
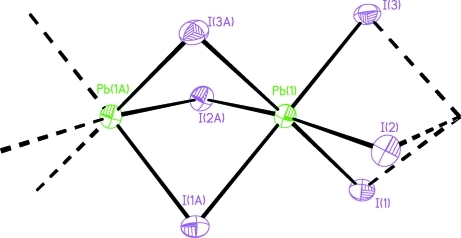
Cut-out of the polymeric polyanion consisting of face-sharing PbI6 octahedra showing displacement ellipsoids at the 30% probability level.

## supplementary crystallographic information

### Comment

Inorganic-organic hybrid materials have attracted intense interest in recent years, owing to their technologically important physical properties from optics to electronics, such as second-order nonlinear optical (NLO) properties, (Guloy *et al.*, 2001) pyroelectricity, ferroelectricity (Horiuchi *et al.*, 2010) and triboluminescence (Chen *et al.*, 2001).

Inorganic metal-halide building blocks exhibiting [*MX*_6_]^4-^/^3-^ fragments (*M* = Sn^2+^, Pb^2+^, Bi^3+^, Sb^3+^; *X* = F^-^, Cl^-^, Br^-^, I^-^) have received special attention in the construction of inorganic-organic hybrid materials (Zhang *et al.*, 2006; Bi *et al.*, 2008). Herein we report the crystal structure of the title compound (I) (Figure 1).

The title compound crystallizes in the orthorhombic space group P*bca* with an asymmetric unit containing one anionic PbI_3_ fragment together with one Schiff base cation. The polymeric anion [PbI_3_]_n_^n-^ possesses slightly distorted PbI_6_ octahedra which are linked to polymeric chains by symmetry related atoms (symmetry code 1/2 - *x*, 1/2 + *y*, *z*). Bond lengths and angles are in good agreement with the other structurally characterized compounds with the same anion (Zhao *et al.*, 2010; Duan *et al.*, 2011)

### Experimental

A mixture of PbI_2_ (461.3 mg, 1.0 mmol) and 1-(2-fluorobenzylideneamino)-quinolinium iodide (377.9 mg, 1.0 mmol) in a 1:1 molar ratio in DMF was slowly evaporated to produce orange-red needle-shaped crystals. The yield of the compound (1) was 67%.

### Refinement

H atoms were placed to the bonded parent atoms in geometrically idealized positions and refined as riding atoms, with *U*_iso_(H) = 1.2*U*_eq_(C).

### Crystal data

**Table:**

(C_16_H_12_FN_2_)[PbI_3_]	*Z* = 8
*M**_r_* = 839.17	*F*(000) = 2976
Orthorhombic, *P**b**c**a*	*D*_x_ = 2.677 Mg m^−^^3^
Hall symbol: -P 2ac 2ab	Mo *K*α radiation, λ = 0.71073 Å
*a* = 20.888 (4) Å	µ = 12.56 mm^−^^1^
*b* = 7.9112 (15) Å	*T* = 296 K
*c* = 25.197 (5) Å	Neddle, orange-red
*V* = 4163.8 (14) Å^3^	0.04 × 0.02 × 0.01 mm

### Data collection

**Table:**

Siemens SMART CCD area-detector diffractometer	4090 independent reflections
Radiation source: fine-focus sealed tube	1770 reflections with *I* > 2σ(*I*)
graphite	*R*_int_ = 0.156
phi and ω scans	θ_max_ = 26.0°, θ_min_ = 1.6°
Absorption correction: multi-scan (*SADABS*; Sheldrick, 2002)	*h* = −25→25
*T*_min_ = 0.747, *T*_max_ = 0.882	*k* = −9→9
30847 measured reflections	*l* = −31→31

### Refinement

**Table:**

Refinement on *F*^2^	Primary atom site location: structure-invariant direct methods
Least-squares matrix: full	Secondary atom site location: difference Fourier map
*R*[*F*^2^ > 2σ(*F*^2^)] = 0.047	Hydrogen site location: inferred from neighbouring sites
*wR*(*F*^2^) = 0.082	H-atom parameters constrained
*S* = 0.96	*w* = 1/[σ^2^(*F*_o_^2^) + (0.0158*P*)^2^] where *P* = (*F*_o_^2^ + 2*F*_c_^2^)/3
4090 reflections	(Δ/σ)_max_ = 0.001
208 parameters	Δρ_max_ = 0.96 e Å^−^^3^
0 restraints	Δρ_min_ = −0.95 e Å^−^^3^

### Special details

**Table:**

Geometry. All e.s.d.'s (except the e.s.d. in the dihedral angle between two l.s. planes) are estimated using the full covariance matrix. The cell e.s.d.'s are taken into account individually in the estimation of e.s.d.'s in distances, angles and torsion angles; correlations between e.s.d.'s in cell parameters are only used when they are defined by crystal symmetry. An approximate (isotropic) treatment of cell e.s.d.'s is used for estimating e.s.d.'s involving l.s. planes.
Refinement. Refinement of *F*^2^ against ALL reflections. The weighted *R*-factor *wR* and goodness of fit *S* are based on *F*^2^, conventional *R*-factors *R* are based on *F*, with *F* set to zero for negative *F*^2^. The threshold expression of *F*^2^ > σ(*F*^2^) is used only for calculating *R*-factors(gt) *etc*. and is not relevant to the choice of reflections for refinement. *R*-factors based on *F*^2^ are statistically about twice as large as those based on *F*, and *R*- factors based on ALL data will be even larger.

### Fractional atomic coordinates and isotropic or equivalent isotropic displacement parameters (Å^2^)

**Table:**

	*x*	*y*	*z*	*U*_iso_*/*U*_eq_	
C12	−0.1124 (8)	0.409 (2)	0.2967 (6)	0.073 (5)	
Pb1	0.24868 (3)	0.30676 (6)	0.394853 (19)	0.04686 (15)	
I1	0.31600 (4)	0.05236 (13)	0.48094 (3)	0.0538 (3)	
I2	0.12891 (4)	0.05643 (12)	0.40030 (4)	0.0583 (3)	
I3	0.31533 (4)	0.05436 (13)	0.30998 (3)	0.0596 (3)	
F1	−0.1508 (4)	0.4781 (12)	0.2589 (4)	0.111 (4)	
N1	0.0224 (5)	0.2690 (14)	0.1530 (4)	0.049 (3)	
N2	0.0119 (5)	0.2585 (14)	0.2088 (4)	0.058 (3)	
C1	−0.0187 (7)	0.2012 (17)	0.1191 (5)	0.060 (4)	
H1	−0.0562	0.1525	0.1318	0.073*	
C2	−0.0062 (7)	0.2018 (18)	0.0630 (6)	0.071 (5)	
H2	−0.0359	0.1579	0.0392	0.086*	
C3	0.0503 (7)	0.2683 (18)	0.0453 (6)	0.064 (4)	
H3	0.0597	0.2675	0.0093	0.077*	
C4	0.0927 (7)	0.336 (2)	0.0803 (7)	0.070 (5)	
C5	0.1517 (7)	0.408 (2)	0.0643 (6)	0.090 (6)	
H5	0.1622	0.4115	0.0284	0.108*	
C6	0.1922 (7)	0.471 (2)	0.0994 (6)	0.112 (7)	
H6	0.2299	0.5216	0.0877	0.134*	
C7	0.1793 (7)	0.464 (2)	0.1555 (6)	0.097 (6)	
H7	0.2087	0.5072	0.1796	0.117*	
C8	0.1249 (7)	0.394 (2)	0.1730 (6)	0.080 (5)	
H8	0.1170	0.3871	0.2092	0.096*	
C9	0.0800 (7)	0.3326 (18)	0.1367 (6)	0.055 (4)	
C10	−0.0397 (6)	0.3288 (15)	0.2223 (5)	0.043 (3)	
H10	−0.0665	0.3773	0.1971	0.052*	
C11	−0.0565 (7)	0.3323 (17)	0.2785 (5)	0.051 (4)	
C13	−0.1289 (7)	0.421 (2)	0.3469 (6)	0.080 (5)	
H13	−0.1660	0.4788	0.3563	0.096*	
C14	−0.0916 (9)	0.350 (2)	0.3848 (7)	0.096 (6)	
H14	−0.1040	0.3526	0.4202	0.115*	
C15	−0.0353 (8)	0.274 (2)	0.3700 (6)	0.083 (6)	
H15	−0.0097	0.2252	0.3959	0.099*	
C16	−0.0158 (7)	0.2680 (18)	0.3182 (6)	0.065 (4)	
H16	0.0238	0.2220	0.3093	0.078*	

### Atomic displacement parameters (Å^2^)

**Table:**

	*U*^11^	*U*^22^	*U*^33^	*U*^12^	*U*^13^	*U*^23^
C12	0.076 (12)	0.102 (14)	0.042 (10)	0.015 (11)	−0.003 (9)	−0.037 (10)
Pb1	0.0469 (3)	0.0437 (3)	0.0499 (3)	−0.0005 (2)	−0.0006 (4)	−0.0024 (3)
I1	0.0494 (6)	0.0675 (6)	0.0444 (5)	−0.0033 (6)	−0.0076 (4)	−0.0005 (5)
I2	0.0422 (5)	0.0616 (6)	0.0710 (6)	−0.0023 (5)	−0.0070 (5)	−0.0082 (6)
I3	0.0676 (6)	0.0681 (6)	0.0432 (5)	−0.0080 (6)	0.0125 (5)	−0.0060 (6)
F1	0.067 (7)	0.161 (10)	0.104 (8)	0.055 (6)	0.000 (6)	−0.021 (7)
N1	0.046 (8)	0.065 (9)	0.035 (8)	0.004 (7)	−0.006 (6)	−0.009 (6)
N2	0.050 (8)	0.080 (9)	0.043 (8)	0.009 (6)	0.010 (6)	−0.005 (6)
C1	0.052 (10)	0.071 (11)	0.059 (11)	0.007 (8)	−0.001 (8)	−0.019 (8)
C2	0.037 (10)	0.117 (15)	0.060 (11)	0.014 (10)	−0.023 (8)	−0.021 (10)
C3	0.044 (10)	0.088 (12)	0.061 (11)	−0.013 (9)	−0.002 (8)	−0.015 (9)
C4	0.039 (10)	0.085 (13)	0.087 (13)	0.008 (9)	0.016 (9)	−0.004 (10)
C5	0.034 (9)	0.190 (19)	0.046 (9)	−0.021 (11)	0.002 (7)	−0.037 (11)
C6	0.059 (11)	0.21 (2)	0.062 (11)	−0.040 (12)	0.019 (10)	−0.037 (13)
C7	0.023 (8)	0.20 (2)	0.066 (11)	−0.017 (11)	0.003 (7)	−0.045 (12)
C8	0.052 (11)	0.124 (16)	0.064 (11)	−0.005 (10)	0.008 (9)	−0.012 (10)
C9	0.043 (10)	0.067 (11)	0.057 (11)	−0.004 (8)	0.013 (8)	−0.013 (9)
C10	0.057 (10)	0.046 (9)	0.027 (8)	0.006 (7)	−0.014 (7)	0.000 (6)
C11	0.044 (9)	0.065 (10)	0.042 (9)	0.001 (8)	0.007 (7)	−0.005 (7)
C13	0.035 (9)	0.134 (15)	0.072 (12)	0.023 (11)	0.016 (8)	−0.017 (12)
C14	0.089 (15)	0.123 (16)	0.077 (15)	0.019 (12)	0.020 (12)	−0.018 (12)
C15	0.071 (14)	0.109 (15)	0.068 (13)	0.014 (11)	−0.011 (10)	0.010 (11)
C16	0.065 (12)	0.095 (13)	0.036 (9)	0.017 (9)	0.005 (9)	−0.004 (9)

### Geometric parameters (Å, °)

**Table:**

C12—C13	1.313 (17)	C3—H3	0.9300
C12—F1	1.359 (16)	C4—C5	1.418 (18)
C12—C11	1.397 (17)	C4—C9	1.446 (18)
Pb1—I3^i^	3.1935 (12)	C5—C6	1.323 (17)
Pb1—I2	3.1938 (11)	C5—H5	0.9300
Pb1—I1^i^	3.2102 (11)	C6—C7	1.440 (18)
Pb1—I2^i^	3.2339 (11)	C6—H6	0.9300
Pb1—I3	3.2402 (11)	C7—C8	1.335 (18)
Pb1—I1	3.2761 (11)	C7—H7	0.9300
I1—Pb1^ii^	3.2102 (11)	C8—C9	1.399 (18)
I2—Pb1^ii^	3.2339 (11)	C8—H8	0.9300
I3—Pb1^ii^	3.1935 (11)	C10—C11	1.457 (16)
N1—C1	1.324 (14)	C10—H10	0.9300
N1—C9	1.366 (15)	C11—C16	1.407 (17)
N1—N2	1.427 (13)	C13—C14	1.355 (19)
N2—C10	1.260 (14)	C13—H13	0.9300
C1—C2	1.437 (17)	C14—C15	1.373 (19)
C1—H1	0.9300	C14—H14	0.9300
C2—C3	1.366 (17)	C15—C16	1.368 (17)
C2—H2	0.9300	C15—H15	0.9300
C3—C4	1.358 (18)	C16—H16	0.9300
			
C13—C12—F1	119.5 (15)	C3—C4—C9	120.7 (15)
C13—C12—C11	124.6 (16)	C5—C4—C9	116.5 (15)
F1—C12—C11	115.9 (13)	C6—C5—C4	121.3 (15)
I3^i^—Pb1—I2	94.65 (3)	C6—C5—H5	119.4
I3^i^—Pb1—I1^i^	84.55 (3)	C4—C5—H5	119.4
I2—Pb1—I1^i^	90.95 (3)	C5—C6—C7	121.4 (15)
I3^i^—Pb1—I2^i^	89.13 (3)	C5—C6—H6	119.3
I2—Pb1—I2^i^	175.06 (4)	C7—C6—H6	119.3
I1^i^—Pb1—I2^i^	86.24 (3)	C8—C7—C6	120.0 (14)
I3^i^—Pb1—I3	96.66 (3)	C8—C7—H7	120.0
I2—Pb1—I3	89.01 (3)	C6—C7—H7	120.0
I1^i^—Pb1—I3	178.79 (3)	C7—C8—C9	120.0 (14)
I2^i^—Pb1—I3	93.71 (3)	C7—C8—H8	120.0
I3^i^—Pb1—I1	179.26 (3)	C9—C8—H8	120.0
I2—Pb1—I1	85.80 (3)	N1—C9—C8	121.5 (14)
I1^i^—Pb1—I1	96.03 (3)	N1—C9—C4	117.6 (14)
I2^i^—Pb1—I1	90.45 (3)	C8—C9—C4	120.8 (15)
I3—Pb1—I1	82.76 (3)	N2—C10—C11	118.4 (12)
Pb1^ii^—I1—Pb1	75.16 (2)	N2—C10—H10	120.8
Pb1—I2—Pb1^ii^	75.97 (2)	C11—C10—H10	120.8
Pb1^ii^—I3—Pb1	75.88 (2)	C16—C11—C12	115.4 (13)
C1—N1—C9	121.7 (13)	C16—C11—C10	122.6 (13)
C1—N1—N2	120.8 (12)	C12—C11—C10	121.9 (13)
C9—N1—N2	117.0 (12)	C12—C13—C14	119.9 (16)
C10—N2—N1	111.8 (11)	C12—C13—H13	120.1
N1—C1—C2	121.0 (14)	C14—C13—H13	120.1
N1—C1—H1	119.5	C13—C14—C15	118.9 (17)
C2—C1—H1	119.5	C13—C14—H14	120.6
C3—C2—C1	118.6 (13)	C15—C14—H14	120.6
C3—C2—H2	120.7	C16—C15—C14	122.0 (16)
C1—C2—H2	120.7	C16—C15—H15	119.0
C4—C3—C2	120.2 (15)	C14—C15—H15	119.0
C4—C3—H3	119.9	C15—C16—C11	119.1 (14)
C2—C3—H3	119.9	C15—C16—H16	120.5
C3—C4—C5	122.9 (16)	C11—C16—H16	120.5

Symmetry codes: (i) −*x*+1/2, *y*+1/2, *z*; (ii) −*x*+1/2, *y*−1/2, *z*.
